# Development of Sequence-Tagged Site Marker Set for Identification of J, J^S^, and St Sub-genomes of *Thinopyrum intermedium* in Wheat Background

**DOI:** 10.3389/fpls.2021.685216

**Published:** 2021-06-23

**Authors:** Linyi Qiao, Shujuan Liu, Jianbo Li, Shijiao Li, Zhihui Yu, Cheng Liu, Xin Li, Jing Liu, Yongkang Ren, Peng Zhang, Xiaojun Zhang, Zujun Yang, Zhijian Chang

**Affiliations:** ^1^College of Agriculture, Shanxi Agricultural University, Taiyuan, China; ^2^Department of Plant Science, College of Agronomy, Northwest Agriculture & Forestry University, Yangling, China; ^3^School of Life Science and Technology, University of Electronic Science and Technology of China, Chengdu, China; ^4^School of Life and Environmental Sciences, Plant Breeding Institute, The University of Sydney, Cobbitty, NSW, Australia; ^5^Department of Botany, College of Life Science, Shanxi University, Taiyuan, China; ^6^School of Life and Environmental Sciences, Plant Breeding Institute, The University of Sydney, Sydney, NSW, Australia

**Keywords:** *Thinopyrum intermedium*, STS markers, specificity, chromosome identification, physical location

## Abstract

*Thinopyrum intermedium* (2*n* = 6*x* = 42, JJJ^S^J^S^StSt) is one of the important resources for the wheat improvement. So far, a few *Th. intermedium* (*Thi*)-specific molecular markers have been reported, but the number is far from enough to meet the need of identifying alien fragments in wheat-*Th. intermedium* hybrids. In this study, 5,877,409 contigs were assembled using the *Th. intermedium* genotyping-by-sequencing (GBS) data. We obtained 5,452 non-redundant contigs containing mapped *Thi*-GBS markers with less than 20% similarity to the wheat genome and developed 2,019 sequence-tagged site (STS) molecular markers. Among the markers designed, 745 *Thi*-specific markers with amplification products in *Th. intermedium* but not in eight wheat landraces were further selected. The distribution of these markers in different homologous groups of *Th. intermedium* varied from 47 (7/12/28 on 6J/6St/6J^S^) to 183 (54/62/67 on 7J/7St/7J^S^). Furthermore, the effectiveness of these *Thi*-specific markers was verified using wheat-*Th. intermedium* partial amphidiploids, addition lines, substitution lines, and translocation lines. Markers developed in this study provide a convenient, rapid, reliable, and economical method for identifying *Th. intermedium* chromosomes in wheat. In addition, this set of *Thi*-specific markers can also be used to estimate genetic and physical locations of *Th. intermedium* chromatin in the introgression lines, thus providing valuable information for follow-up studies such as alien gene mining.

## Introduction

*Thinopyrum intermedium* (Host) Barkworth & D.R. Dewey (2*n* = 6*x* = 42, JJJ^S^J^S^StSt) belongs to the tribe Triticeae, which is a perennial cross-pollinated species and cultivated as a forage grass worldwide (Vogel and Jensen, 2001). It is also an ideal species for water and soil conservation and saline–alkali land improvement (Li and Wang, 2009). It is generally believed that the *Th. intermedium* J sub-genome is partially homologous to the genomes of *Th. bessarabicum* (2*n* = 2*x* = 14, J^b^J^b^) and *Th. elongatum* (2*n* = 2*x* = 14, J^e^J^e^), the St sub-genome is contributed by *Pseudoroegneria spicata* (2*n* = 2*x* = 14, StSt), whereas the J^S^ sub-genome is derived from the J sub-genome partially recombined with the St genome (Chen et al., 1998; Mahelka et al., 2013).

*Th. intermedium* can be readily hybridized with common wheat (*Triticum aestivum* L., 2*n* = 6*x* = 42, AABBDD) (Peto, 1936; Stebbins and Pun, 1953; Dewey, 1984; Li et al., 2015). It has excellent quality, stress tolerance, and disease resistance, especially against powdery mildew, rusts, barley yellow dwarf virus, and wheat streak mosaic virus (Chang et al., 2010; Bao et al., 2014; Salina et al., 2015; Li et al., 2016; Zhang et al., 2020), making it an important wild resource for the wheat improvement. In the 1960s, Tsitsin (1965) obtained the wheat-*Th. intermedium* octoploid for the first time through distant hybridization, which initiated the exploration and utilization of *Th. intermedium*. Thereafter, *Th. intermedium* chromosomes have been introgressed into wheat, resulting in the production of wheat-*Th. intermedium* addition, substitution, and translocation lines (Forster et al., 1987; Chen et al., 1999; Yang et al., 2006; Zhan et al., 2015; Li et al., 2017). It is critical in germplasm enhancement to identify alien chromatin by genomic *in situ* hybridization (GISH) (Chen et al., 1998, 1999; Chen, 2005) or fluorescence *in situ* hybridization (FISH) (Chang et al., 2010; Li et al., 2015, 2016; Salina et al., 2015; Zhang et al., 2020). At present, several FISH probes, such as Oligo-pDb12H derived from *Dasypyrum villosum* (Yu et al., 2019) and Oligo-B11 and Oligo-pThp3.93 from *Th. ponticum* (Xi et al., 2019), are used to differentiate *Th. intermedium* chromosomes from wheat chromosomes. Recently, a set of pooled oligo probes Synt1~7 was developed to distinguish the seven homologous groups (HGs) of Triticeae species including *Th. intermedium* (Li et al., 2021). However, for very small alien fragments in the later generation of hybrids, it is difficult to determine their positions in the *Th. intermedium* genome cytologically; therefore, the identification based on molecular markers is crucial.

Markers from wheat or rice (*Oryza sativa* L.), such as simple sequence repeat (SSR) markers, PCR-based landmark unique gene (PLUG) markers, and single-nucleotide polymorphism (SNP) markers, were used as complementary means of cytological identification to detect alien fragments in wheat genome (Chen, 2005; Bao et al., 2010; Li et al., 2017, 2021; Xi et al., 2019; Yu et al., 2019). The SNP genotyping array (Cseh et al., 2019) and kompetitive allele-specific PCR (KASP) genotyping assay (Grewal et al., 2020) can also be used for characterizing wheat-*Th. intermedium* introgression lines. However, the position of the introgressed alien fragments in the *Th. intermedium* genome cannot be determined.

It is particularly important to develop specific markers directly based on the *Th. intermedium* sequences. In 2016, Kantarski et al. (2017) explored genotyping-by-sequencing (GBS) markers in *Th. intermedium* and constructed the first consensus genetic map containing all *Th. intermedium* linkage groups (*Thi*-LG1~21) using seven genetic populations. However, the sub-genome information corresponding to each *Thi*-LG remains unknown. Subsequently, Wang R. R. C. et al. (2020) compared the GBS sequences of *Ps. spicata* with the previously released *Thi*-GBS sequences and identified *Thi*-LG2, 4, 8, 11, 13, 17, and 21 as the St sub-genome. In this study, the above-mentioned *Thi-*GBS sequences were compared with the annotated coding sequence (CDS) data of *Th. elongatum* published recently (Wang H. et al., 2020) to distinguish the J and J^S^ sub-genomes in *Thi*-LGs. Then, contigs assembled with the original *Thi*-GBS sequences were selected to develop sequence-tagged site (STS) markers. The *Thi*-specific markers that have amplification products in *Th. intermedium* but not in common wheat were identified, thereby providing an economical and convenient tool for identifying *Th. intermedium* fragments in wheat.

## Materials and Methods

### Plant Materials

Six independent plants from the same *Th. intermedium* accession (in order to avoid the individual differences caused by cross-pollination) and eight wheat landraces (in order to avoid the possibility that wheat cultivars may contain alien species fragments such as 1B/1R, which will affect the screening results) from different ecological regions in China were used to screen the *Thi*-specific markers. *Th. elongatum, Th. bessarabicum, Ps. Spicata*, and *D. villosum* were used as the related species of *Th. intermedium* to detect the amplification of these *Thi*-specific markers. Wheat-*Th. intermedium* partial amphidiploids, addition lines, substitution lines, and translocation lines were used to test the effectiveness of the *Thi*-specific markers. Materials used in this study and their relevant information including name, genome composition, and providers are listed in Table 1.

**Table 1:** Plant materials used in this study.

**Line**	**2*n*=**	**Genomic formula**	**Accession**	**Provider**
*Thinopyrum intermedium*	42	JJ^S^St	Z1141	Current laboratory
*Triticum aestivum*	42	ABD	Chinese Spring	a
	42	ABD	Shanglinxiaomai	
	42	ABD	Louguding	
	42	ABD	Xiaobaimang	
	42	ABD	Chadianhong	
	42	ABD	Jiangxizao	
	42	ABD	Lanhuamai	
	42	ABD	Motuoxiaomai	
*Th. elongatum*	14	J^e^	PI 531717	b
*Th. bessarabicum*	14	J^b^	PI 610232	c
*Pseudoroegneria spicata*	14	St	PI 499493	b
*Dasypyrum villosum*	14	V	PI 610786	c
Partial amphiploid	56	ABD+1J+2St+3J+4St+5J+6St+7J	TAF46	d Forster et al., 1987; Friebe et al., 1992; Chen et al., 1999
	56	ABD+1St+2J^S^+3J+4J+4J^S^+5J^S^+6St+7St	TE-3	d Yang et al., 2006; Hu et al., 2011; Song et al., 2013; Li et al., 2015, 2017, 2019
Addition line	44	ABD+2J^S^	X24C14	d Li et al., 2017
	44	ABD+3J	A1082	d Li et al., 2019
	44	ABD+4St	L4	d Forster et al., 1987; Chen et al., 1999
Substitution line	42	ABD+1St (1D)	AS1677	d Hu et al., 2011
	42	ABD+4J (4B)	X24C10	d Li et al., 2017
	42	ABD+4J^S^ (4B)	A1125	d
	42	ABD+6J^S^ (6B)	XM-4	d
Translocation line	42	ABD+T4BS/4JL	T1332	d
	42	ABD+T4BS.5J^S^L	A39	d
	44	ABD+T7J^S^S/3AS.3AL+T7J^S^S.7J^S^L/3AL	Z4	d Lang et al., 2018, e

a *Millet Research Institute, Shanxi Agricultural University, Changzhi, Shanxi, China. The wheat landraces used in this study were from different ecological regions of China. Chinese Spring: southwestern winter wheat region; Shanglinxiaomai: south China winter wheat region; Louguding: Yellow and Huai River Valleys winter wheat region; Xiaobaimang: northern spring wheat region; Chadianhong: northern winter wheat region; Jiangxizao: middle and lower Yangtze valley winter wheat region; Lanhuamai: northwestern spring wheat region; Motuoxiaomai: Qinghai-Tibet spring-winter wheat region*.

b *Germplasm Bank of Triticeae Research Institute, Sichuan Agricultural University, Chengdu, Sichuan, China*.

c *Crop Research Institute, Shandong Academy of Agricultural Sciences, Ji'nan, Shandong, China*.

d *School of Life Science and Technology, University of Electronic Science and Technology of China, Chengdu, Sichuan, China*.

e *Plant Breeding Institute, The University of Sydney, Cobbitty, NSW, Australia*.

### Informatics Analysis of *Thi*-GBSs

The method used to distinguish sub-genomes in *Thi*-LGs was described by Wang R. R. C. et al. (2020). The 10,029 *Thi*-GBS sequences mapped to *Thi*-LG1~21 (Kantarski et al., 2017) were aligned with the annotated CDSs of *Th. elongatum* (accession number GWHABKY00000000, version 1.0) (Wang H. et al., 2020) obtained from the National Geophysical Data Center database (NGDC, https://bigd.big.ac.cn/) with BLAST tool (version 2.6.0+), setting E ≤ 1.0 ×10^−25^. For the *Thi*-GBS sequences with multiple hits, the hit with the lowest e-value was selected for further analysis. In the same HG of *Th. intermedium*, the *Thi*-LG with the most matched *The*-CDSs was presumed to be the J sub-genome. For the number of significant hits, a Chi-squared test was performed with the Bonferroni adjustment for multiple tests to determine if observed values were significantly different.

### Sequence Assembly and Primer Design

The original *Thi*-GBS data (accession number SRX3008333) downloaded from the Sequence Read Archive database (https://www.ncbi.nlm.nih.gov/sra/) was assembled as contigs using the SOAPdenovo2 software^1^ (Luo et al., 2012). After removing redundancy, contigs containing the mapped *Thi*-GBS marker (Kantarski et al., 2017) were used to blast the wheat genome (cv. Chinese Spring, version 1.0) downloaded from the International Wheat Genome Sequencing Consortium database (IWGSC, https://urgi.versailles.inra.fr/) (Lukaszewski et al., 2014). Then, the contigs with sequence similarity of less than 20% were obtained for developing STS markers. A Primer 3.0 software-based^2^ script written by the Perl language (Han et al., 2015) was used for a large-scale primer design, and the parameters were set as following: primer length was 18–22 bp, and the product length was 100–400 bp.

### Screening and Validation of the *Thi*-Specific Markers

The developed STS markers were tested on six *Th. intermedium* individuals and eight wheat landraces, and those that can amplify in *Th. intermedium* but not in wheat were selected as the *Thi*-specific markers. These markers were then used on wheat-*Th. intermedium* partial amphiploids, addition lines, and substitution lines to verify their effectiveness. In addition, the amplification results of the *Thi*-specific markers in *Th. bessarabicum, Th. elongatum, Ps. spicata*, and *D. villosum* were visualized by the Venn diagram (http://bioinformatics.psb.ugent.be/webtools/Venn/) and were subjected to the phylogenetic analysis using MEGA6.0^3^ (Tamura et al., 2013) with the neighbor-joining method and 1,000 bootstraps. A physical location of *Thi*-specific markers was obtained by blasting against the genome data of *Th. intermedium* (version 2.1, http://phytozome.jgi.doe.gov/).

PCR was performed in 10 μl reaction using PCR Mix (B532061, Sangon Biotech, Shanghai, China). Amplified products were electrophoresed in 8% non-denaturing polyacrylamide gels and then stained in a 0.1% silver nitrate solution.

### Fluorescence *in situ* Hybridization and GISH Analyses

Mitotic metaphase chromosomes were obtained from root tips and were spread according to the procedures as described in Lang et al. (2018). Four oligo-nucleotide probes, such as Oligo-pSc119.2, Oligo-pTa535 (Tang et al., 2014), Oligo-k288 (Wang et al., 2019), and Oligo-B11 (Kantarski et al., 2017), were used to identify wheat and *Th. intermedium* chromosomes. They were 5′-end labeled with either 6-carboxyfluorescein (6-FAM) for green signals or 6-carboxytetramethylrhodamine (Tamra) for red signals (Supplementary Table 1). The protocol of non-denaturing FISH (ND-FISH) using oligo probes was according to Fu et al. (2015). The FISH images were captured with an Olympus BX-51 Microscope equipped with a DP-70 CCD Camera (Shinjuku, Tokyo, Japan) or a Zeiss Axio Imager Microscope (Oberkochen, Germany) equipped with a Retiga EXi CCD Camera (QImaging, Surrey, BC, Canada).

After stripping off the oligo probes, the same slides were analyzed by GISH as described in Zhang et al. (2001). Total genomic DNA from *Th. intermedium* (Cytogenetic stock accession C05.05, University of Sydney) was labeled with biotin-16-dUTP (Roche Diagnostics Australia, Castle Hill, NSW, Australia) using nick translation. Unlabeled total genomic DNA of wheat was used as a blocker. The probe to blocker ratio was ~1:80. Signals were detected with Fluorescein Avidin DN (Vector Laboratories, Burlingame, CA, USA). Chromosomes were counterstained with DAPI and pseudo-colored red.

## Results

### Determination of Sub-genomes for *Thi*-GBS Sequences

Using blastn of homology analysis, 284 *The*-CDSs were matched at a minimum e-value of 1.0 ×10^−25^ with those in 10,029 *Thi*-GBS sequences reported previously (Kantarski et al., 2017) (Table 2). Because the J^S^ genome incorporated part of the St genome (Chen et al., 1998; Mahelka et al., 2013), the similarity between the *The-*J^e^ genome and the *Thi*-J sub-genome is higher than that between the *The-*J^e^ genome and the *Thi*-J^S^ sub-genome. Therefore, *Thi*-LGs 1, 6, 7, 10, 14, 18, and 20 can be confidently assigned to *Thi*-HGs 1, 2, 3, 4, 5, 6, and 7 of the J sub-genome, respectively (Table 2). Because *Thi*-LGs 2, 4, 8, 11, 13, 17, and 21 were reported as *Thi*-HGs 1, 2, 3, 4, 5, 6, and 7 of the St sub-genome (Wang R. R. C. et al., 2020), respectively, the remaining seven *Thi*-LGs 3, 5, 9, 12, 15, 16, and 19 were presumed to be the 1J^S^−7J^S^ sub-genomes.

**Table 2:** Locations of 284 *The*-CDSs matched with *Thi*-GBS sequences reported in Kantarski et al. (2017).

***The*-HGs**	***Thi*****-HG1**			**-HG2**			**-HG3**			**-HG4**			**-HG5**			**-HG6**			**-HG7**			**Total**	**% in expected HG**
	LG1	2	3	4	5	6	7	8	9	10	11	12	13	14	15	16	17	18	19	20	21		
1J^e^	20^a^	9	10																			39	51
2J^e^				10	11	24^a^								1								46	52
3J^e^							19^a^	6	7		1		1							1	1	36	53
4J^e^										18^a^	6	5	1		2							32	56
5J^e^						1				1		2	6	37^a^	7					1		55	67
6J^e^											1					6	4	16^a^				27	59
7J^e^								1											9	27^a^	12	49	55
	1J	1St	1J^S^	2St	2J^S^	2J	3J	3St	3J^S^	4J	4St	4J^S^	5St	5J	5J^S^	6J^S^	6St	6J	7J^S^	7J	7St		
Total																						284	

a *These numbers are significantly different from the two other observations within the same homologous group (HG) at p ≤ 0.007 level (Bonferroni corrected)*.

### Development of *Thi*-Specific Markers

**Figure 1:**
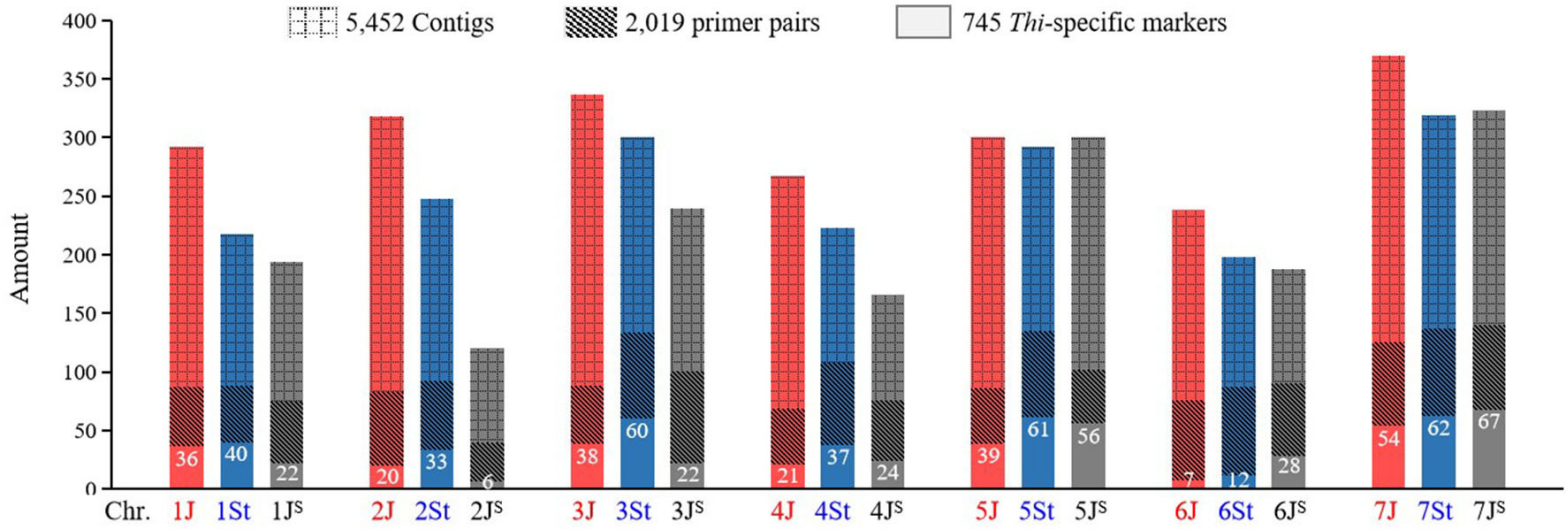
Amount and distribution of the GBS-contigs and markers developed in *Th. intermedium*. The number of *Thi*-specific markers on each chromosome is listed.

A total of 5,877,409 contigs were assembled using the original *Thi*-GBS sequences, ranging in length from 100 to 3,094 bp, with a total length of 915,311,073 bp (Supplementary Table 2). After removing the redundancy, 5,452 contigs containing the mapped *Thi*-GBS markers (Kantarski et al., 2017) were identified. In total, 2,019 STS markers were developed for the 5,452 non-redundant contigs, with 250, 215, 323, 253, 323, 253, and 402 markers distributed in the *Thi*-HG1 to HG7, respectively (Figure 1).

**Figure 2:**
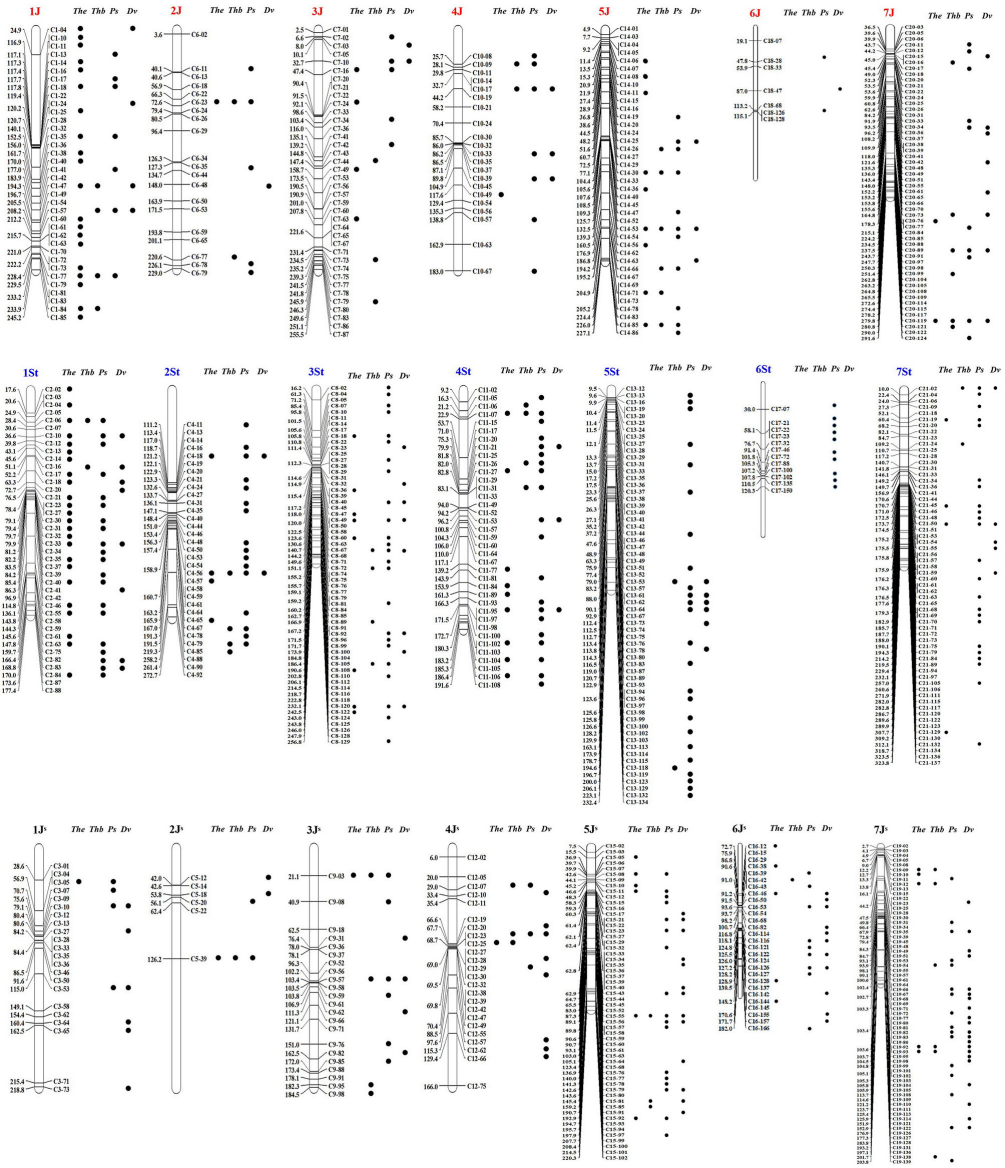
A linkage map of 745 *Thi*-specific STS markers derived from the *Thi*-GBS linkage map reported in Kantarski et al. (2017). Black dots next to the markers indicate that these markers have amplification products in *Th. elongatum* (*The*), *Th. bessarabicum* (*Thb*), *Ps. spicata* (*Ps*), and *D. villosum* (*Dv*).

Out of the 2,019 STS markers, 745 amplified only in *Th. intermedium* but failed in the eight wheat landraces were considered as the *Thi*-specific markers (Supplementary Figure 1 and Supplementary Table 3). Linkage maps are shown in Figure 2, and the marker distribution in *Thi*-HG1-7 was 98 (36/40/22, 1J/1St/1J^S^), 59 (20/33/6, 2J/2St/2J^S^), 120 (38/60/22, 3J/3St/3J^S^), 82 (21/37/24, 4J/4St/4J^S^), 156 (39/61/56, 5J/5St/5J^S^), 47 (7/12/28, 6J/6St/6J^S^), and 183(54/62/67, 7J/7St/7J^S^). Among them, 224, 306, and 233 markers were located respectively in the J, St, and J^S^ sub-genome.

### Evaluation of *Thi*-Specific Markers Using Wheat-*Th. intermedium* Lines

**Figure 3:**
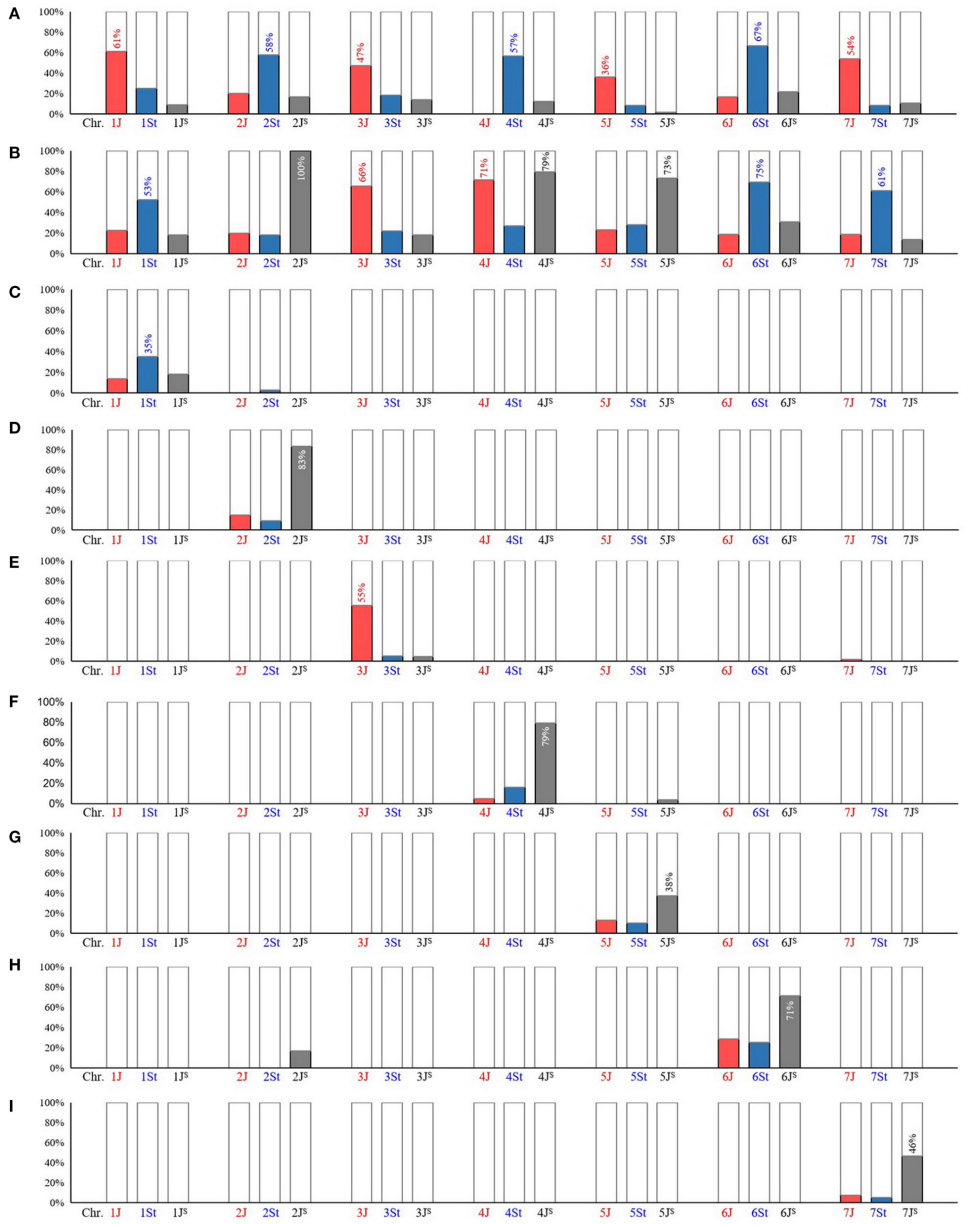
Amplification of *Thi*-specific markers in wheat-*Th. intermedium* introgression lines. **(A)** partial amphiploid TAF46 (ABD+1J+2St+3J+4St+5J+6St+7J); **(B)** partial amphiploid TE-3 (ABD+1St+2J^s^+3J+4J+4J^s^+5J^s^+6St+7St); **(C)** substitution line AS1677 [ABD+1St(1D)]; **(D)** addition line X24C14 (ABD+2J^s^); **(E)** addition line A1082 (ABD+3J); **(F)** substitution line A1125 [ABD+4J^s^(4B)]; **(G)** translocation line A39 (ABD+T4BS.5J^s^L); **(H)** substitution line XM-4 [ABD+6J^s^(6B)]; **(I)** translocation line Z4 (ABD+T7J^s^S-3AS.3AL+T7J^s^S.7J^s^L-3AL). The sub-genomes with the largest proportion of positive markers were labeled, and the numbers on the column were significantly different from the two other observations within the same homologous group (HG) at *p* ≤ 0.0125 level (Bonferroni corrected).

The *Thi*-specific markers were used to amplify two wheat-*Th. intermedium* partial amphiploids TAF46 (ABD+1J+2St+3J+4St+5J+6St+7J) and TE-3 (ABD+1St+2J^s^+3J+4J+4J^s^+5J^s^+6St+7St). The detectable rate of *Thi*-specific markers in 1J (61%), 2St (58%), 3J (47%), 4St (57%), 5J (36%), 6St (77%), and 7J (54%) was higher than that of other sub-genomes in the corresponding HG in TAF46 (Figure 3A). In TE-3 (Figure 3B), the sub-genomes with high detectable rate were 1St (53%), 2J^S^ (100%), 3J (66%), 4J (71%), 4J^s^ (79%), 5J^s^ (73%), 6St (69%), and 7St (61%).

**Figure 4:**
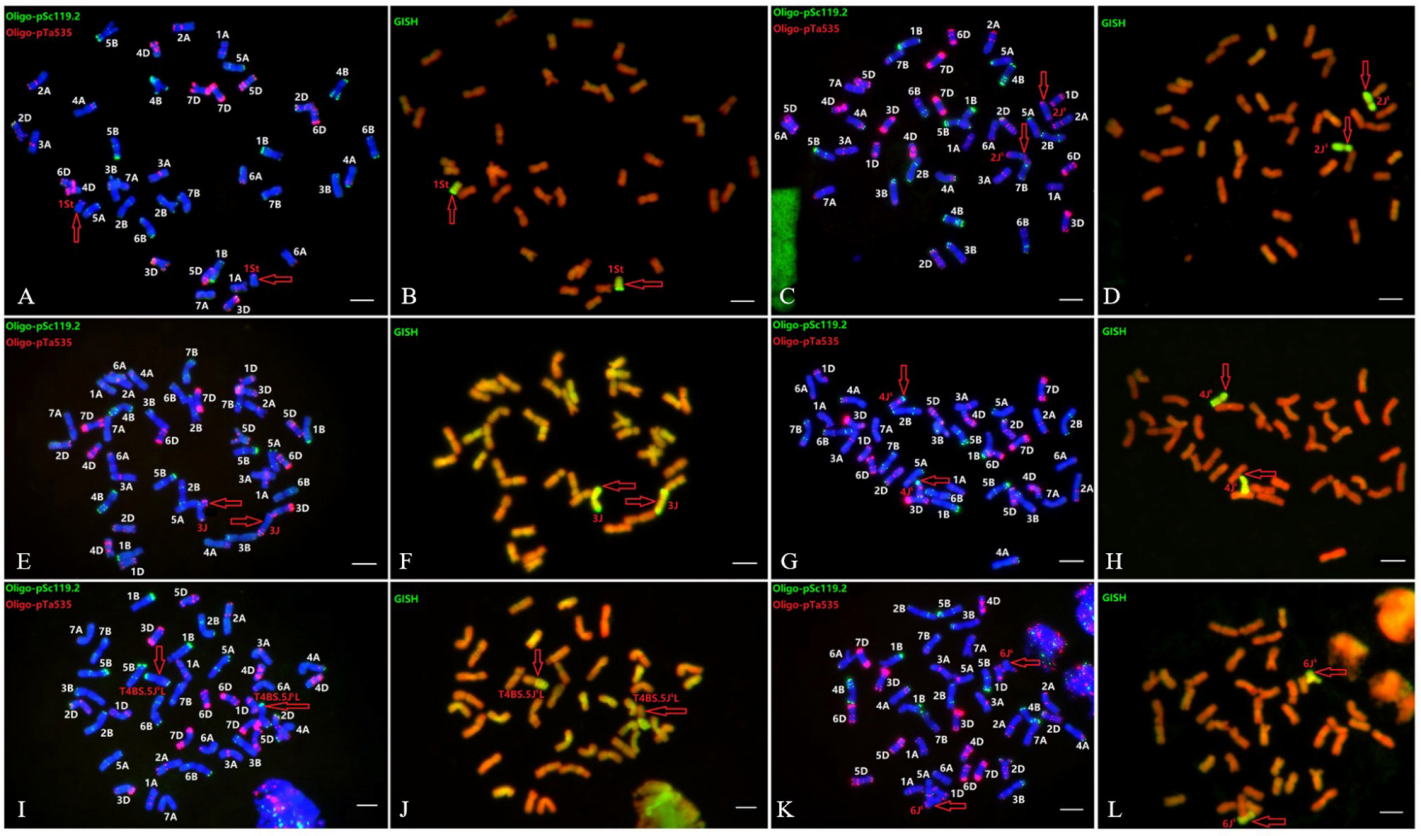
Sequential FISH and GISH patterns of six wheat-*Th. intermedium* introgressions with single alien sub-genome from *Thi*-HG1-6, respectively. **(A,B)** substitution line AS1677 [ABD+1St(1D)]; **(C,D)** addition line X24C14 (ABD+2J^s^); **(E,F)** addition line A1082 (ABD+3J); **(G,H)** substitution line A1125 [ABD+4J^s^(4B)]; **(I,J)** translocation line A39 (ABD+T4BS.5J^s^L); and **(K,L)** substitution line XM-4 [ABD+6J^s^(6B)]. The probes for FISH were Oligo-pSc119.2 (green) + Oligo-pTa535 (red) **(A,C,E,G,I,K)**. The probe (yellow-green) for GISH analysis was *Th. intermedium* total genomic DNA **(B,D,F,H,J,L)**. Bars, 10 μm.

Furthermore, six wheat-*Th. intermedium* introgressions with single alien sub-genome from different *Thi*-HGs were characterized, namely AS1677 [ABD+1St(1D)], X24C14 (ABD+2J^S^), A1082 (ABD+3J), A1125 [ABD+4J^S^(4B)], A39 (ABD+T4BS.5J^S^L), and XM-4 [ABD+6J^S^(6B)] (Figure 4). These six introgressions and Z4 (ABD+T7J^S^S-3AS.3AL+T7J^S^S.7J^S^L-3AL) (Lang et al., 2018) were further used to evaluate the *Thi*-specific markers, which were able to detect the introgressed *Thi*-chromosomes in these lines correctly (Figures 3C–I), exhibiting good specificity among *Thi*-HGs. However, there was a certain degree of non-specificity in distinguishing the three sub-genomes within the same *Thi*-HG.

### Prediction of the Positions in of Alien Segments *Th. intermedium* by *Thi*-Specific Markers

**Figure 5:**
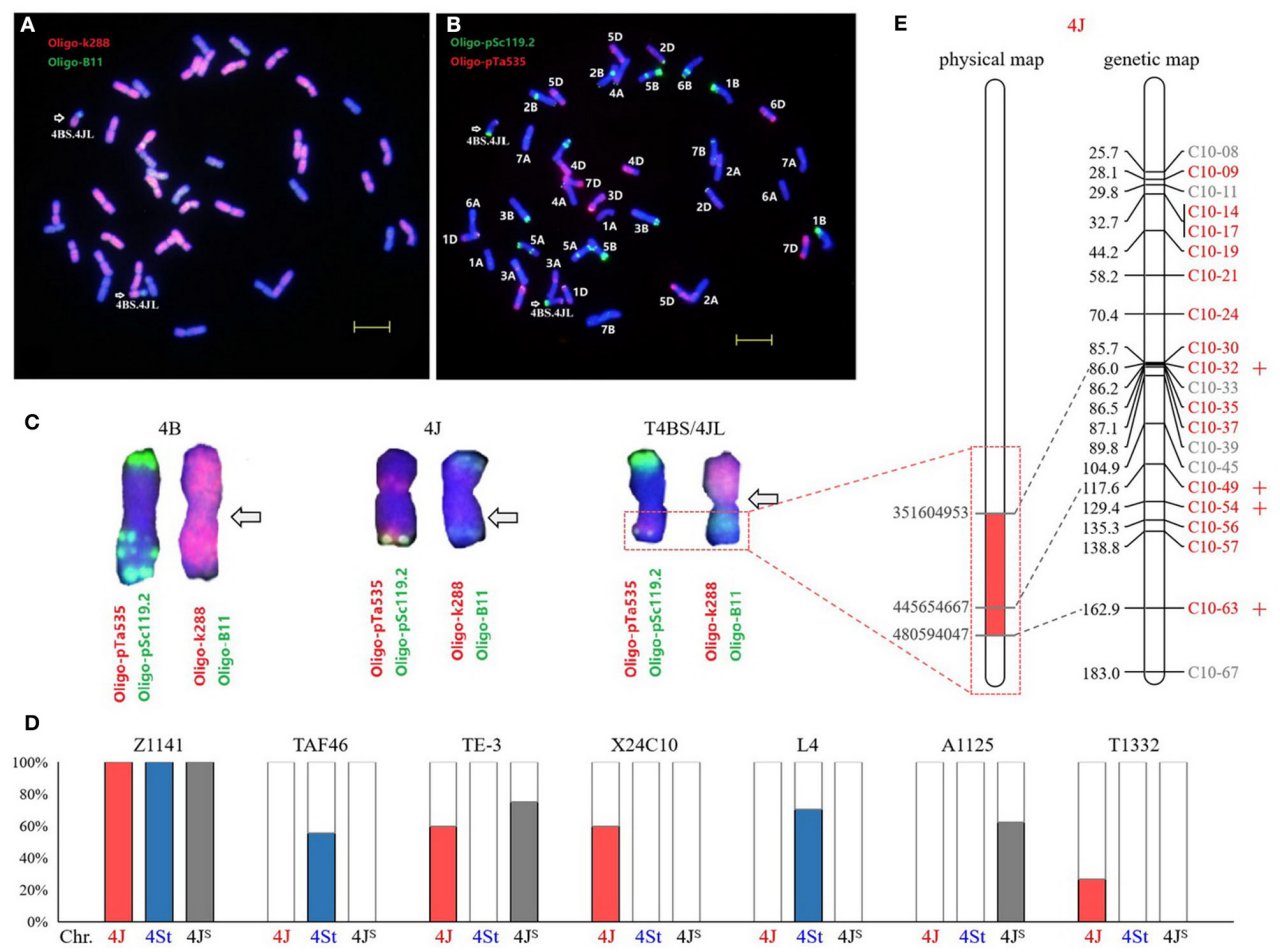
Identification of wheat-*Th. intermedium* translocation line T1332. The probes were Oligo-k288 (red) + Oligo-B11 (green) **(A)** and Oligo-pSc119.2 (green) + Oligo-pTa535 (red) **(B)**. Bars, 10 μm. The chromosomes in the karyotype **(C)** were 4B, 4J, and T4BS/4JL, respectively, and the arrows pointed to the translocation breakpoints. **(D)** Amplification of 4J-, 4St-, and 4J^s^-specific markers in *Th. intermedium* and six wheat-*Thi* introgression lines. **(E)** The position of 21 *Thi*-specific markers on 4J physical and linkage maps. Red markers are 4J-specific, four of which with amplification products in translocation line T1332 are marked with plus sign.

The *Thi*-specific markers were used to predict the positions of *Th. intermedium* chromatin in T1332, a translocation line introduced segment of the long arm of *Thi-*chromosome 4J (Figures 5A–C). In order to improve the chromosome specificity of markers, 82 *Thi*-specific markers of *Thi*-HG4 were used on the substitution line X24C10 with *Thi*-chromosome 4J (4B) (Li et al., 2017) and the 4St addition line L4 (Forster et al., 1987; Chen et al., 1999). Combined with the previous identification results in the substitution line A1125 4J^S^ (4B) (Figure 3F) and two partial amphiploids TAF46 and TE-3 (Figures 3A,B), 58 (71%) *Thi*-chromosome-specific markers were identified, of which 15 were 4J-specific, 27 were 4St-specific, and 16 were 4J^S^-specific (Figure 5D). Among the 58 *Thi*-chromosome specific markers in T1332 (ABD+T4BS/4JL) showed that four 4J-specific markers C10-32, C10-49, C10-54, and C10-63 amplified target products. According to the physical location of these markers, it could be inferred that the introduced fragment contained the chromosome interval 4J:351604953-480594047Mb of *Th. intermedium* (Figure 5E).

### Amplification of *Thi*-Specific Markers in the Je/Jb/St/V Genomes

**Figure 6:**
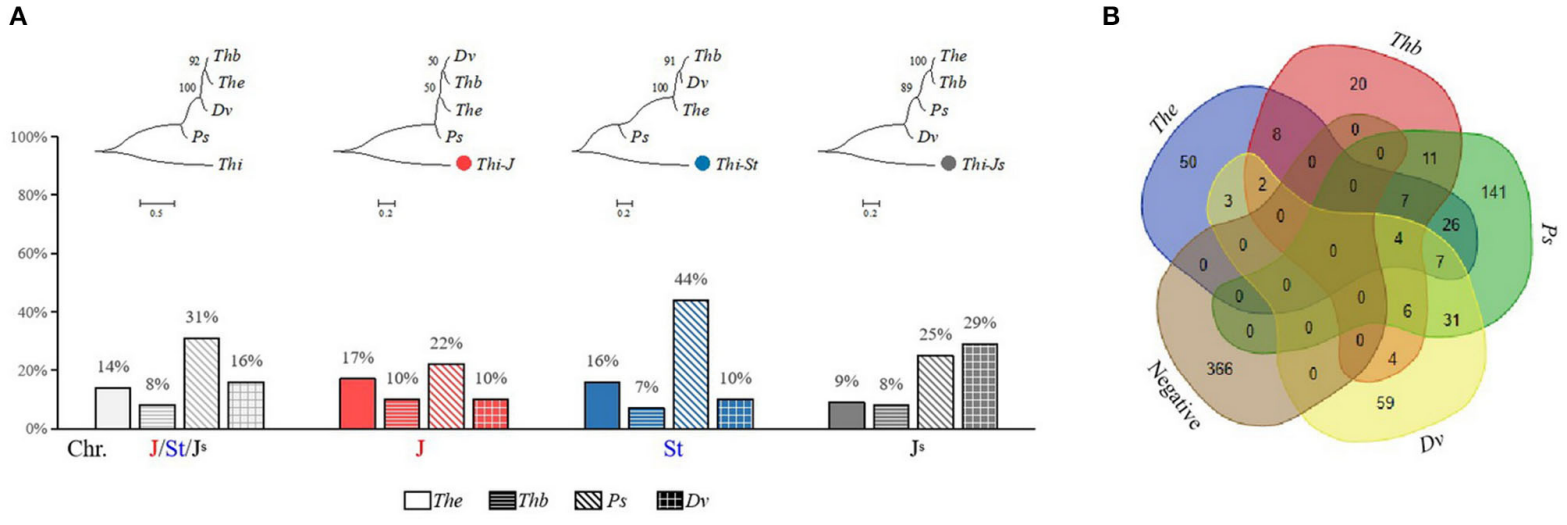
Amplification of *Thi*-specific markers in the J^e^/J^b^/St/V genomes. **(A)** Phylogenetic analysis and proportion of the positive *Thi*-specific markers in *Th. elongatum* (*The*), *Th. bessarabicum* (*Thb*), *Ps. spicata* (*Ps*), and *D. villosum* (*Dv*). **(B)** Number of the negative and positive *Thi*-specific markers in *Th. elongatum, Th. bessarabicum, Ps. spicata*, and *D. villosum*.

The amplification results of *Thi*-specific markers showed that 107 (14%), 62 (8%), 233 (31%), and 116 (16%) markers could be amplified in *Th. elongatum, Th. bessarabicum, Ps. spicata*, and *D. villosum*, respectively. Among them, the markers located in the J and St sub-genomes were amplified the most in *Ps. spicata*, 22 and 44%, respectively, whereas markers in the J^S^ sub-genome were amplified the most in *D. villosum* (29%) (Figure 6A). Similarly, the phylogenetic analysis showed that the J and St sub-genomes were closely related to *Ps. spicata*, whereas the J^S^ sub-genome is relatively close to *D. villosum* (Figure 6A). The number of *Thi*-specific markers that specifically amplify in *Th. elongatum, Th. bessarabicum, Ps. spicata*, and *D. villosum* were 50, 20, 141, and 59, respectively, whereas 366 markers were not amplified in the above species (Figure 6B).

## Discussion

*Thinopyrum intermedium* is one of the important resources for the wheat improvement. In this study, 2,019 STS markers distributed on 21 *Thi*-chromosomes were developed based on the *Thi*-GBS sequences and used to amplify from *Th. intermedium* and eight wheat landraces from different ecological regions in China. Many species polymorphisms, including the presence or absence or the length difference of amplicons, were obtained. In order to identify the *Thi*-specific fragments in the wheat background more accurately, 745 *Thi*-specific markers with amplicons in *Th. intermedium* but not in wheat were screened. Due to the homology among the three sub-genomes J/J^S^/St of *Th. intermedium*, the developed *Thi*-specific markers are not exclusively specific to the corresponding sub-genome within the same HG. Using wheat-*Th. intermedium* introgression lines, 58 out of 82 (71%) *Thi*-specific markers in *Thi*-HG4 were identified. However, due to the lack of materials with single *Thi*-sub-genome introgressed, the *Thi*-specific markers in the remaining *Thi*-HGs were not identified.

We used this set of markers to accurately identify the alien chromosomes derived from different *Thi*-HGs in the wheat-*Th. intermedium* addition, substitution, and translocation lines. Chromosome 1St, 2J^S^, 3J, 4J, and 7J have been reported to carry genes for stripe rust resistance (Hu et al., 2011; Li et al., 2017, 2019; Lang et al., 2018). In addition, chromosome 4J also carries genes related to dwarf, tillering, and blue grain (Li et al., 2017). We will then identify whether the introgression lines have obtained beneficial agronomic traits from *Th. intermedium* and use them to develop small-fragment translocation lines. The *Thi*-specific markers will be used to track alien fragments and determine the approximate chromosomal location of the target alien gene.

The set of *Thi*-specific markers developed in this study can be used to identify not only *Th*. *intermedium* chromosomes in the wheat background, but also the alien chromosomes from other Triticeae species with J and St genomes, such as *Th. ponticum* (2*n* = 10*x* = 70, JJJJ^S^J^S^/E^e^E^b^E^x^StSt) (Zhang et al., 1996, 2001; Chen et al., 1998). Some *Thi*-specific markers can amplify species-specific bands in *Th. elongatum, Th. bessarabicum, Ps. spicata*, and *D. villosum*. Therefore, these markers can also be suitable for the identification of the alien chromosomes from the above species in the wheat background.

There are several advantages of this set of markers identified in this study. First, they are PCR-based markers, which are easy to use and cost-effective. Second, this set of markers, covering all *Th. intermedium* chromosomes, are developed based on the GBS markers from the published *Th. intermedium* genetic map, so each *Thi*-specific marker has a corresponding map location. Third, they can be used for the chromosome identification after further screening, whereas the current SNP chip and KASP chip cannot accurately identify the J and J^S^ sub-genomes, which is due to the high similarity between the J and J^S^ sub-genomes and the characteristic duality of SNP (Cseh et al., 2019; Grewal et al., 2020). Fourth, the physical positions of *Thi*-specific markers in *Th. intermedium* can be determined according to their contigs, so the sequence of small alien fragment in wheat-*Th. intermedium* translocations can be inferred, which can provide valuable information for further identification of small alien fragments, and even for the cloning of alien genes.

However, this set of markers also has some limitations. For substitution lines and translocation lines, it is impossible to identify which wheat chromosomes have been replaced or translocated onto. Therefore, cytological techniques or wheat chromosome-specific markers are needed for the identification. In addition, the distributions of *Thi*-specific markers on certain chromosomes are insufficient (such as chromosomes 2J^S^, 6J, and 6St, Figure 2) or uneven (such as chromosomes 1J and 2St, Figure 2). Thus, the alien *Thi*-segments that are not covered by markers cannot be detected. Furthermore, for wheat varieties with complex genetic backgrounds, especially containing multiple alien fragments, the accuracy of this set of markers will be affected.

Due to cross-pollination, genetic exchange between *Th. intermedium* and other species may occur, resulting in a complex evolutionary process and genome composition of *Th. intermedium*. Since 1936, several genome constitutions of *Th. intermedium*, such as AXY (Peto, 1936), BEF (Stebbins and Pun, 1953), B_2_X_1_X_2_ (Dewey, 1984), J^r^J^vs^St (Cseh et al., 2019), have been proposed. At present, it is generally believed that the genome constitution of *Th. intermedium* should be JJ^S^St based on the GISH results with the St, J, and E genomic DNA probes, among which the St sub-genome is thought to derive from *Ps. spicata*, whereas the origins of J and J^S^ sub-genomes are still uncertain (Chen et al., 1998; Mahelka et al., 2013). Studies have shown that these sub-genomes are partial homology with *Th. elongatum, Th. bessarabicum*, and *D. villosum*, in which the J^S^ sub-genome is also partially recombined with the St genome (Chen et al., 1998; Mahelka et al., 2011, 2013). In this study, 44% of the markers located in the St sub-genome were positive in *Ps. spicata*, which was much higher than that in *Th. elongatum* (16%), *Th. bessarabicum* (7%), and *D. villosum* (10%), indicating that *Thi*-St genome has good homology with *Ps. spicata* genome. The markers of J^S^ sub-genome have a high amplification percentage in *D. villosum* (29%) and *Ps. spicata* (25%), which is consistent with the reported GISH results (Mahelka et al., 2011). However, there were also many positive markers from the J sub-genome in *Ps. spicata* (22%), indicating the complexity of the origin of J sub-genome. In *Th. elongatum*, the percentage of positive *Thi*-J markers (17%) was higher than that of the positive *Thi*-J^S^ markers (9%), which also confirms the feasibility of using *The*-CDSs to distinguish the J sub-genome from the 21 *Thi*-LGs.

As many as 366 (49%) of *Thi*-specific markers failed to amplify in *Th. elongatum, Th. bessarabicum, Ps. spicata*, and *D. villosum*, indicating that the *Th. intermedium* genome has undergone extensive recombination and gradually evolved into a new species after polyploidization by natural hybridization, which is a common phenomenon in nature (Hegarty and Hiscock, 2005). Moreover, there is a possibility that except for the above four species, there may be other species involved in the evolution of *Th. intermedium*, such as *Aegilops tauschii* (D genome) and *Taeniatherum* (Ta genome) (Mahelka et al., 2011). Therefore, the negative *Thi-*specific markers can be applied to other Triticeae species, which may be able to discover species close to the *Th. intermedium* genome or involved in the evolution of *Th. intermedium*.

## Data Availability Statement

The original contributions presented in the study are included in the article/Supplementary Material, further inquiries can be directed to the corresponding author/s.

## Author Contributions

ZYa and LQ designed the experiments. LQ developed the STS markers. ZYa and PZ provided the wheat-*Th. intermedium* introgression lines. SLiu, SLi, and JLiu performed the PCR experiments. JLi, ZYu, and PZ conducted the GISH and FISH experiments. CL, XL, and YR helped with data analysis. LQ and JLi wrote the manuscript. PZ, ZYa, XZ, and ZC revised the manuscript. All authors contributed to the article and approved the submitted version.

## Conflict of Interest

The authors declare that the research was conducted in the absence of any commercial or financial relationships that could be construed as a potential conflict of interest.

## Footnotes

^1^https://github.com/aquaskyline/SOAPdenovo2.

^2^https://sourceforge.net/projects/primer3/.

^3^https://www.megasoftware.net/.
