# Supplementary material for: Development of Sequence-Tagged Site Marker Set for Identification of J, JS, and St Sub-genomes of Thinopyrum intermedium in Wheat Background
Source: Front Plant Sci. 2021 Jun 23;12:685216. doi: 10.3389/fpls.2021.685216 (PMC8261300; doi:10.3389/fpls.2021.685216)
Supplement: Supplementary file 1 [file Data_Sheet_1.docx]

Supplementary data


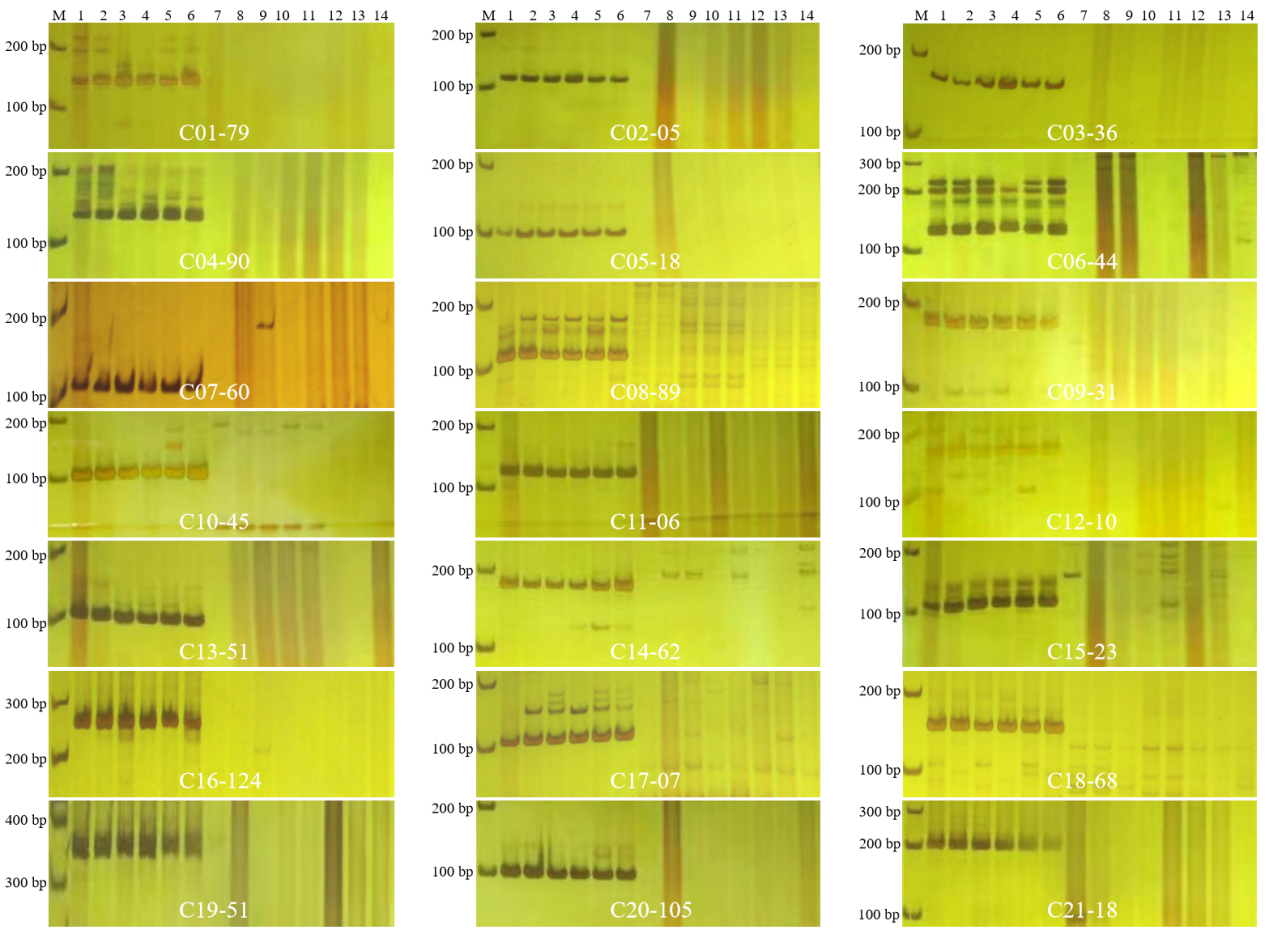


**SUPPLEMENTARY FIGURE S1 | Polyacrylamide gel images showing amplification results of 21 *Thi*-specific markers on each *Thi*-chromosome in six *Th. intermedium* plants and eight wheat landraces.** M: DNA ladder; C01-C21: markers for all 21 *Th. intermedium* chromosomes (1J-7St); 1-6: six *Th. intermedium* plants; 7-14: Chinese Spring, Shanglinxiaomai, Louguding, Xiaobaimang, Chadianhong, Jiangxizao, Lanhuamai, Motuoxiaomai.
